# Optimization of CRISPR–Cas system for clinical cancer therapy

**DOI:** 10.1002/btm2.10474

**Published:** 2022-12-23

**Authors:** Xiang Meng, Tian‐gang Wu, Qiu‐yue Lou, Kai‐yuan Niu, Lei Jiang, Qing‐zhong Xiao, Tao Xu, Lei Zhang

**Affiliations:** ^1^ College & Hospital of Stomatology Anhui Medical University, Key Laboratory of Oral Diseases Research of Anhui Province Hefei People's Republic of China; ^2^ Anhui Provincial Center for Disease Control and Prevention Hefei People's Republic of China; ^3^ Clinical Pharmacology, William Harvey Research Institute (WHRI), Barts and The London School of Medicine and Dentistry Queen Mary University of London (QMUL) Heart Centre (G23) London UK; ^4^ Department of Otolaryngology The Third Affiliated Hospital of Anhui Medical University Hefei China; ^5^ School of Pharmacy, Anhui Key Laboratory of Bioactivity of Natural Products Anhui Medical University Hefei China; ^6^ Inflammation and Immune Mediated Diseases Laboratory of Anhui Province Hefei China; ^7^ Department of Periodontology Anhui Stomatology Hospital Affiliated to Anhui Medical University Hefei China

**Keywords:** cancer, CRISPR–Cas, gene editing, synthetic biology, vector delivery

## Abstract

Cancer is a genetic disease caused by alterations in genome and epigenome and is one of the leading causes for death worldwide. The exploration of disease development and therapeutic strategies at the genetic level have become the key to the treatment of cancer and other genetic diseases. The functional analysis of genes and mutations has been slow and laborious. Therefore, there is an urgent need for alternative approaches to improve the current status of cancer research. Gene editing technologies provide technical support for efficient gene disruption and modification in vivo and in vitro, in particular the use of clustered regularly interspaced short palindromic repeats (CRISPR)–Cas systems. Currently, the applications of CRISPR–Cas systems in cancer rely on different Cas effector proteins and the design of guide RNAs. Furthermore, effective vector delivery must be met for the CRISPR–Cas systems to enter human clinical trials. In this review article, we describe the mechanism of the CRISPR–Cas systems and highlight the applications of class II Cas effector proteins. We also propose a synthetic biology approach to modify the CRISPR–Cas systems, and summarize various delivery approaches facilitating the clinical application of the CRISPR–Cas systems. By modifying the CRISPR–Cas system and optimizing its in vivo delivery, promising and effective treatments for cancers using the CRISPR–Cas system are emerging.

**Abbreviations**
AML: acute myelogenous leukemia
AAVs: adeno‐associated viruses
AVs: adenoviruses
BP: black phosphorus
CPPs: cell‐penetrating peptides
CRISPR: clustered regularly interspaced short palindromic repeats
crRNAs: CRISPR RNAs
CTLs: cytotoxic T lymphocytes
DSB: double‐strand breaks
EVs: extracellular vesicles
FRL: far‐infrared light
AuNPs: gold nanoparticles
GO: graphene oxide
HSC: hematopoietic stem cell
hgRNA: homing guide RNA
HDR: homology‐directed repair
hTERT: human telomerase reverse transcriptase
hUP II: human uroplakin II gene
LVs: lentiviruses
LAN: lance array nanoinjection
NIR: near‐infrared light
NHEJ: nonhomologous end joining
PC: pancreatic cancer
PDAC: pancreatic ductal adenocarcinoma
PEI: polyethyleneimine
PAM: protospacer adjacent motif
PFS: protospacer flanking site
RNP: ribonucleoprotein
scRNA: scaffold RNA
shRNA: short hairpin RNA
SNMs: single nucleotide mutations
sgRNA: single‐stranded guide RNA
SMA: spinal muscular atrophy
ThT: thiaxanthin T
TALENs: transcription activator‐like effector nucleases
UCNPs: upconversion nanoparticles
ZFNs: zinc‐finger nucleases

## 1 INTRODUCTION

Cancer is one of the most important health problems worldwide. It was estimated that there were 19.3 million new cases and 10 million cancer deaths worldwide in 2020.^1^ Cancer remains the leading disease‐associated cause of death, despite rapid advances in treatments.^2^ Traditional therapies suffer from disadvantages such as poor specificity and resistance to chemotherapy drugs.^3^ The exact mechanisms underlying the poor effectiveness of traditional therapies are not yet clear. Thus, uncovering the genetic pattern of cancer and subsequently deepening the understanding of its role in cancer development and response to treatment has become a major focus of attention.^4^

The availability of zinc‐finger nucleases (ZFNs) and transcription activator‐like effector nucleases (TALENs) offers the possibility of directly targeting and modifying genomic sequences.^5^, ^6^ However, ZFNs and TALENs are expensive and inefficient, limiting their clinical application.^7^ In this aspect, clustered regularly interspaced short palindromic repeats and CRISPR‐associated genes (CRISPR–Cas) system offers promising solutions to such limitations, with multiple advantages.^8^ Yan et al.^9^ used CRISPR/Cas9 to knock down miR‐3064 and showed that the proliferation, invasion, and tumorigenic ability of pancreatic cancer (PC) cells were significantly inhibited. Meanwhile, CRISPR–Cas system could define vulnerabilities in cancer by identifying essential genes, gene interactions, and anticancer immune targets.^10^ Importantly, the advent of multiple toolkits further extended the application of the CRISPR–Cas systems in cancer.

In this review, we conducted an in‐depth analysis of the mechanism of action of the CRISPR–Cas systems, and the research progress of newly emerging toolboxes to modify and deliver CRISPR systems. We also highlighted the potential added effects of incorporating synthetic biology into the CRISPR–Cas systems for cancer treatment. We further explored the delivery issues currently faced by the CRISPR–Cas system for clinical studies, with the hope of further refining the CRISPR–Cas systems to facilitate clinical application.

## 2 THE COMPONENTS AND MECHANISMS OF CRISPR–Cas SYSTEMS

**FIGURE 1:**
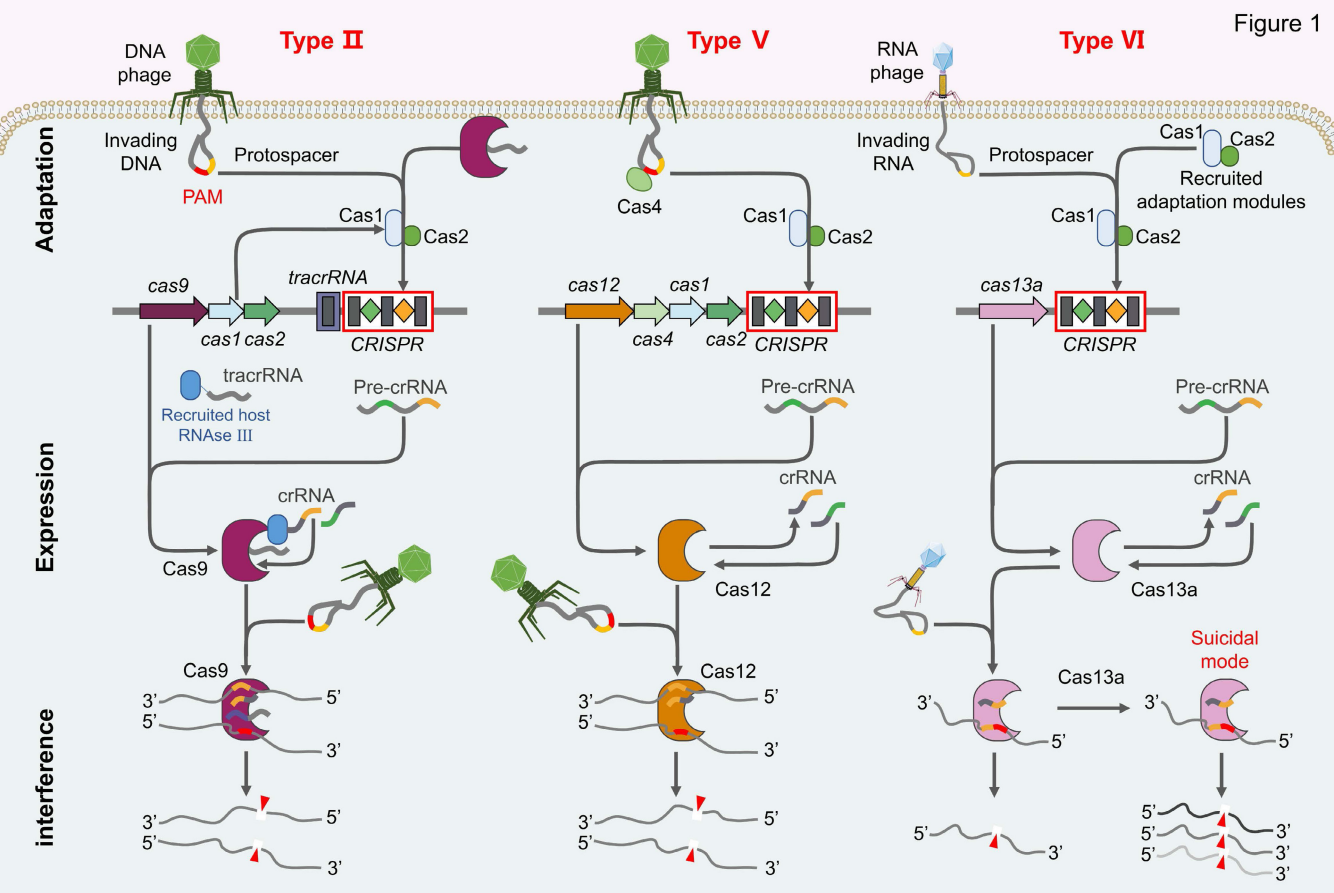
Invasive phages trigger adaptive immune mechanisms of the Class II CRISPR–Cas systems in bacteria and archaea. Both mechanisms of type II and type V are triggered by DNA phages, whereas type VI is triggered by RNA phages. All three types of mechanism are divided into three steps: adaptation, expression, and interference. Acquisition of spacer sequences requires Cas1 and Cas2 proteins. Targeting of dsDNA produces interference activity in the case of type II and V systems, but targeting of ssRNA in the case of type VI system. In the classical type II system, tracrRNA is processed by RNase III, after which the tracrRNA, Cas9, and RNase III complexes process the transcribed CRISPR array (pre‐crRNA). The type II effector complex consisting of Cas9, tracrRNA, and guide crRNA cleaves the target DNA to produce flat ends

**FIGURE 2:**
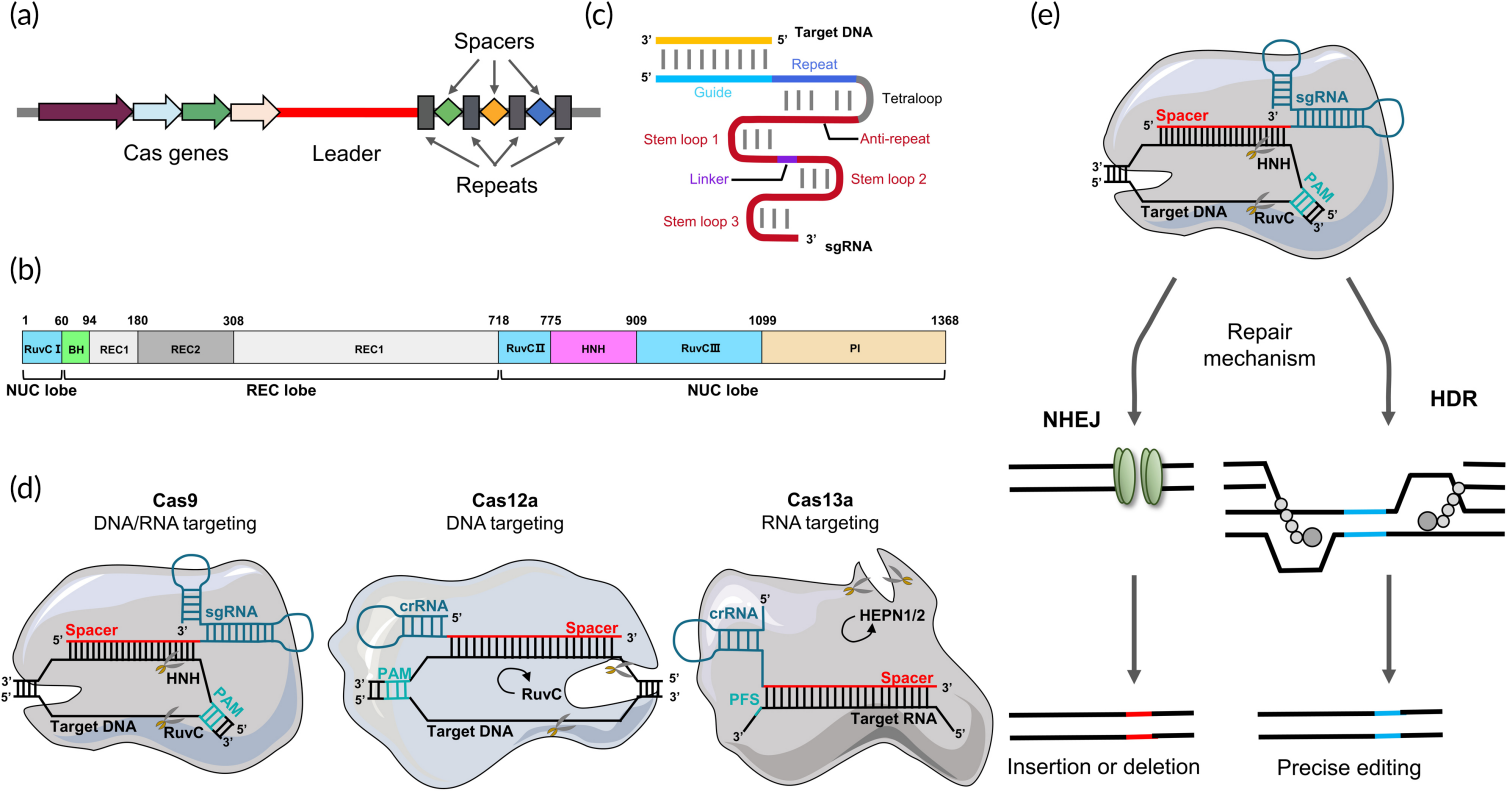
The structure of the Class II CRISPR–Cas systems and gene editing mechanism (with CRISPR–Cas9 as an example). (a) Typical structure of CRISPR locus. The CRISPR gene sequence is mainly composed of the leader, repeats and spacers. The leader sequence is located upstream of the CRISPR gene and is considered as the promoter of the CRISPR sequence. The repeats are about 20–50 bp base length and the transcription products can form hairpin structures. The spacers are exogenous DNA sequence that are captured by the bacteria. (b) The domain organization of SpCas9 consists of NUC lobe and REC lobe. BH, Bridge helix. (c) Schematic representation of the sgRNA:target DNA complex. Artificially designed target sequences of sgRNAs function as crRNA‐tracRNA complexes, which can direct Cas9 proteins to specifically cleave target genes. (d) Schematic representation of representative Cas proteins from different families (shown are Cas9, Cas12a, and Cas13a). In CRISPR–Cas9, the sgRNA‐encoded spacer binds to the target dsDNA near the PAM. Base pairing activates the HNH and RuvC nuclease structural domains, which separate the two strands. In CRISPR–Cas12a, the crRNA‐encoded spacer binds to the target base and activates the RuvC nuclease, cleaving both strands with multiple‐turnover general ssDNase activity (arrow). In CRISPR–Cas13a, the target sequence is RNA. Correct base‐pairing activates HEPN nuclease general ssRNase activity (arrow). (e) Genome editing using CRISPR–Cas9. The Cas9 nuclease binds to the sgRNA, which in turn is directed to the target DNA by complementary base pairing. PAM sequence (NGG, NAG) must be present in the anterior segment of the target sequence. Cleavage of the next double‐stranded DNA (dsDNA) triggers the error‐prone nonhomologous end joining (NHEJ) or homologous directed repair (HDR) mechanism

The CRISPR–Cas systems, consisting of CRISPR arrays and highly diverse Cas genes, are adaptive immune systems evolved by bacteria and archaea in their immune system against invading phages and foreign plasmid DNA^11^, ^12^ (Figure 1). Structurally, CRISPR arrays contain a leader (adjoining the first repeat of CRISPR loci and considered as the promoter of CRISPR arrays), short direct repeats (forming hairpin structures to stabilize the secondary structure of RNA), and nonrepetitive spacers (captured exogenous DNA sequences)^13^, ^14^ (Figure 2a). These arrays can be transcribed and processed into CRISPR RNAs (crRNAs), which are used to direct Cas nucleases to cleave complementary exogenous DNA sequences.^15^ At least 45 natural Cas proteins have been identified in different bacteria, as exemplified by the well‐known *Streptococcus thermophilus* (St1) has three Cas genes: Cas9, Cas1, and Cas2.^16^ The domain organization of SpCas9 consists of NUC lobe and REC lobe (Figure 2b). Meanwhile, depending on the architecture of the CRISPR array and the signature interference effector, CRISPR–Cas systems can be classified into two classes [containing six types (I–VI) and 33 sub‐types].^17^, ^18^ Class I systems (including type I, III, and IV) encompass multisubunit Cas effector proteins, which bind to crRNA and generate target interference. Additionally, class II systems (including type II, V, and VI) require only a single, multidomain large Cas effector protein to form a complex with crRNA in the interference process.^19^ Accordingly, the class II systems represented by CRISPR–Cas9 require only one Cas effector protein to function as a cleavage,^20^ while the class I systems demand multiple Cas effector proteins, limiting their applications.^21^ Hence, class II systems exhibit tremendous promises for genome engineering in cleavage of target DNA and RNA.

Simply put, bacteria and archaea are able to store a small segment of viral gene (named spacer) into the CRISPR array when they are first invaded by a virus. When the same virus invades again, the bacteria are able to recognize the virus based on the spacer and disable it by cutting off the DNA of the virus. The specific process involves three major steps (using CRISPR–Cas9 as an example): adaptation, expression, and interference. The adaptation stage is the spacer acquisition to form memory of previous infections and is what makes CRISPR–Cas immunization adaptive and heritable. Spacer acquisition relies on Cas1 and Cas2 which are present in almost all CRISPR–Cas systems.^18^ Cas1 is catalytic and Cas2 has a structural function.^22^ Adaptation mechanisms show a preference for foreign DNA over self‐DNA as the key to avoiding autoimmunity. For example, the RecBCD repair complex present in *Escherichia coli* is able to degrade larger portions of the foreign genome and serves as the basis for preferential access to nonself DNA.^23^ Once the exogenous DNA is injected into the host, the proteins encoded by Cas1 and Cas2 would recognize protospacer adjacent motif (PAM) in the exogenous DNA sequence, and then take DNA sequence adjacent to the PAM as a protospacer.^24^ Next, the Cas1/2 protein complex snips the protospacer from exogenous DNA to form a spacer which is inserted between two repeats at the 5′‐end of CRRSPR array with the assistance of enzymes.^25^, ^26^

At the expression stage, CRISPR array (repeats and spacers) is transcribed to generate pre‐crRNA (crRNA precursor) and tracrRNA (trans‐activating CRISPR RNA) under the initiation of leader. Pre‐crRNA is sheared into mature crRNA (containing 1 spacer and several repeats) by RNase III nuclease and Cas9 protein. Repeats and tracrRNA form a double‐stranded RNA (tracrRNA‐crRNA) by base complementary pairing which assembles into a complex with Cas9 protein.^27^ The effector complex composed of Cas9 and the tracrRNA:crRNA duplex exerts interference after a second cleavage by an unknown RNase—which removes the 5′ repeat‐derived tag.^28^ The spacer is in free state. This complex would monitor exogenous DNA sequence at all times.^29^

During the interference stage, the Cas9 effector protein is already bound to the guide RNA prior to target selection and cleavage, thus participating in crRNA maturation. The spacer recognizes the complementary sequence in exogenous DNA. The entire complex is also localized to specific PAM, and the DNA double‐strand is then unraveled. The spacer sequence hybridizes to the complementary strand, while the other strand remains free. Subsequently, Cas9 protein is localized to the correct PAM sequence.^30^ Base pairing of crRNA with the target strand induces an R‐loop structure that eventually triggers cleavage of the target and nontarget strands by the domains of Cas9 protein, respectively, resulting in flat‐end cleavage at three nucleotides upstream of the PAM.^28^ This eventually results in DNA double‐strand breaks (DSB), silencing of exogenous DNA expression, and successful immunization.^31^ Thus, the interference protects hosts from invasion of exogenous genome and also gives a chance for gene editing since typical CRISPR–Cas9 system brings a break of double‐stranded DNA.

It can be seen that CRISPR array is used to identify exogenous DNA sequences, while Cas9 protein acts as scissors for cleavage. Among them, tracrRNA–crRNA serves as the navigator of the system and guides Cas9 for precise targeting. Importantly, in 2012, Jinek et al.^32^ designed a single‐stranded guide RNA (sgRNA) that replaced the crRNA–tracrRNA complex, which can direct the Cas9 protein to specifically cleave the target gene (Figure 2c). This success confirms the feasibility of artificially designed sgRNAs for target sequence by means of synthetic biology, thus enabling gene repair and modeling of mutations, knock‐out, knock‐in, fusion.^33^ Especially in recent years, the rise of multiple Cas proteins has once again brought the advantages of CRISPR–Cas systems to the forefront with remarkable achievements.

## 3 COMMONLY USED CAS PROTEINS AND THEIR NOVEL DERIVATIVES

Although many CRISPR–Cas systems have been identified, only a few of them have been used as research tools. Among them, class II systems relying on a single‐effector Cas protein are widely used for gene editing in mammals. Specifically, the representative effector proteins in the class II are Cas9 (type II), Cas12 (type V), and Cas13 (type VI)^34^, ^35^, ^36^ (Figure 2d). The characteristics of the different Cas proteins can be seen in Table 1.

**TABLE 1:** Features of naturally occurring major CRISPR–Cas enzymes

Type/class	Effector	Nuclease domains	Function	Size (amine acids)	Molecular weight (kDa)	PAM sequence	Length of guiding sequence (bp)	Cutting site
II/2	spCas9	RuvC, HNH	DNA nuclease	1368	158.3	NGG	20	~3 bp 5′ of PAM
	FnCas9	RuvC, HNH	DNA nuclease	1628	190.3	NGG	20	~3 bp 5′ of PAM
	SaCas9	RuvC, HNH	DNA nuclease	1052	123.8	NNGR R	21	~3 bp 5′ of PAM
	NmCas9	RuvC, HNH	DNA nuclease	1081	124.2	NNNNG ATT	24	~3 bp 5′ of PAM
	St1Cas9	RuvC, HNH	DNA nuclease	1122	129.5	NNAGA AW	20	~3 bp 5′ of PAM
	St3Cas9	RuvC, HNH	DNA nuclease	1409	164.9	NGGNG	20	~3 bp 5′ of PAM
	CjCas9	RuvC, HNH	DNA nuclease	983	114.8	NNNNACAC	22	~3 bp 5′ of PAM
V/2	AsCpf1	RuvC	crRNA processing, DNA nuclease	1307	151.2	TTTV	24	19/24 bp 3′ of PAM
	LbCpf1	RuvC	crRNA processing, DNA nuclease	1228	143.7	TTTV	24	19/24 bp 3′ of PAM
VI/2	Cas13	HEPN × 2	crRNA processing, RNA nuclease	Multiple orthologs	Variable	RNA targeting	28	/

### 3.1 Cas9

Cas9, the most widely used effector protein, has two structural domains with cleavage activity: the HNH domain (responsible for cleaving the complementary DNA strand with crRNA) and the Ruvc domain (responsible for cleaving the noncomplementary DNA strand).^37^ The commonly used Cas9 protein is derived from *Streptococcus pyogenes* (SpCas9), whose PAM sequence is NGG (N is any nucleotide). To further extend the diversity of PAM sequences, over 10 different Cas proteins have been identified in the last few years. For example, the smallest Cas9 nuclease is from *Campylobacter jejuni* (CjCas9), which has only 984 amino acids and its PAM sequence is NNNNNACAC. The small size of CjCas9 allows for good intracellular delivery, but its targeting range and flexibility are relatively limited.^38^

Cas9 nuclease cleaves DNA and produces genome editing effects via nonhomologous end joining (NHEJ) or homology‐directed repair (HDR) pathways (Figure 2e). A variety of Cas9 mutation systems have been developed to address the off‐target effects of CRISPR–Cas9. In 2013, Ran et al. mutated one of the two catalytically active domains of Cas9 (HNH or RuvC) to obtain Cas9 nickase (Cas9n).^39^ This form of mutation produces single‐stranded gaps rather than double‐stranded breaks, and allows gene editing via the HDR pathway. If double‐stranded DNA needs to be cleaved, the two gRNAs will be designed to be on opposite DNA strands and in close proximity (sequences no more than 20 bp apart), thus effectively introducing a DSB. Ultimately, on‐target stringency can be increased while off‐target mutations are minimized with Cas9n.^40^ Further, by mutating both nuclease active regions of Cas9, a dead Cas9 (dCas9) that specifically recognizes only sgRNA and has no shearase activity was generated.^41^ dCas9 is mainly fused with transcriptional regulatory elements or chromosomal modification elements to build new tools for the regulation of transcriptional and epigenetic modifications such as CRISPRa (transcriptional activation), CRISPRi (transcriptional interference), and CRISPRoff (controlling gene expression with high specificity while leaving the DNA unchanged).^42^, ^43^, ^44^ For example, dCas9 promotes or represses the transcription of target genes by binding to activation domains (VP16, VP64, NF‐κB) or repression domains (KRAB, MIX1).

To further overcome the targeting limitations of PAM, Walton et al.^45^ designed new Cas9 variants, named as SpG and SpRY that bind and cleave DNA without specific PAM and are capable of unrestricted targeting the majority of the human genome with single base‐pair precision. It is thus clear that the off‐target and PAM sequence defects of the CRISPR–Cas9 system can be refined, leaving the efficient delivery of CRISPR–Cas9 as the remaining obstacle for in vivo application of the CRISPR–Cas9 systems, which will be addressed in a subsequent section. Overall, optimized CRISPR–Cas9 system can be applied to a wider range of fields, including gene therapy for cancer.

### 3.2 Cas12

Unlike Cas9, Cas12 nuclease contains only a RuvC‐like domain that cleaves two strands to induce DSB.^46^ Since possessing RNAase and DNAase activity, the Cas12 nuclease relies on a single crRNA guide for DNA localization and cleaves at the distal PAM end to produce 5‐nt sticky ends, in contrast to Cas9 which normally cleaves near the PAM end to produce blunt ends.^47^ Widely used Cas12a (known as Cpf1) is from *Acidaminococcus* spp. (AsCas12a) and *Lachnospiraceae* spp. (LbCas12a), with a small molecular mass of 1200 to 1300 amino acids.^48^ On the one hand, unlike the G‐rich PAMs required for Cas9, Cas12a can recognize T‐rich PAMs, thus further increasing the number of potential target sites. On the other hand, Cas12a can follow its own cleavage pattern and PAM sequences to generate staggered ends, facilitating precisely targeted integration of DNA. The restricted recognition of PAM (5′‐TTTN‐3′) by AsCas12a and LbCas12a limits their application in the field of gene editing.^49^ An enhanced AsCas12a variant (enAsCas12a) has been designed to improve genome editing activity. Meanwhile, the targeting range for Cas12a has been expanded greatly by newly engineered AsCas12a variants that recognize PAMs 5′‐TYCV and 5′‐TATV, or PAMs 5′‐VTTV, 5′‐TTTT, 5′‐TTCN, and 5′‐TATV.^50^, ^51^ Another reported Cas12a with a *Francisella novicida* origin (FnCas12a) has a PAM sequence of 5′‐KYTV‐3′ (K is T and G; Y is C and T; V is A, C, and G) and possesses DNA cleavage activity in human cells at multiple loci.^52^ The extended PAM sequences enhance selectable regions of target sites and enrich applications of Cas12a in gene editing.

Cas12a is not only flexible but also shows a high degree of specificity. Kim et al.^53^ used Digenome‐seq to analyze the whole genome after the action of different gene‐editing enzymes. It was found that for the same crRNA, LbCas12a and AsCas12a had 6 and 12 off‐target sites, respectively, which were far fewer than those caused by Cas9 (>90 sites). Moreover, Kleinstiver et al.^54^ compared the off‐targets of AsCas12a and LbCas12a with those of SpCas9 in vivo using GUIDE‐seq analysis. The results showed that Cas12a had a mutation frequency as low as ~0.1%–0.2% in the majority of off‐target sites, indicating that Cas12a has very limited off‐targets compared to SpCas9. Similar to Cas9, by mutating the RuvC domain, a catalytically inactive version of Cas12a (dCas12a) was generated.^55^ dCas12a is able to bind different enzymes, thereby mediating transcriptional activation. For example, potent transcriptional activation of dLbCas12a can be achieved by fusing synthetic activation complexes consisting of VP16, p65, and the Rta activator domains.^56^ The SunTag system is known to contain multiple scFv fusions to VP64, where scFv is a single‐chain variable fragment antibody against GCN4.^57^ The dLbCas12a gene can be efficiently activated by binding SunTag to the C‐terminus of dLbCas12a.^58^ However, transcriptional activation was only observed with dLbCas12a, whereas dAsCas12a induced merely marginal activation.^58^

The smaller size of Cas12a facilitates intracellular delivery. Also, PAM distal cleavage ensures the safety of target recognition of sgRNAs by the small indels generated through the NHEJ repair pathway. Importantly, Cas12a has been demonstrated to have high specificity and low off‐target effects. Consequently, emerging new Cas derivatives including Cas12b, Cas12c, Cas12d (CasY), Cas12e (CasX), Cas12g, Cas12h, Cas12i, and Cas12j (CasΦ),^59^, ^60^, ^61^, ^62^ will greatly expand gene‐editing adaptations in tumor therapy.

### 3.3 Cas13

Both Cas9 and Cas12a have been shown to target DNA, but no single Cas effector protein can target RNA. In 2016, Shmakov et al.^20^ identified a new Cas effector protein named as C2c2 (Cas13a) that can target RNA. Subsequently, Liu et al.^63^, ^64^ and Knott et al.^65^ reported that Cas13a contains two HEPN domains with RNase activity. Since then, another three Cas13 family proteins were identified, namely Cas13b, Cas13c, and Cas13d.^66^, ^67^ Cas13a is a two‐component system with the binding domain of the catalytic site on the outer surface of the protein, which performs cis or trans cleavage of RNA.^64^ The Cas13b system lacks Cas1 and Cas2 and is capable of targeting the secondary structure of RNA.^68^ Cas13d is 20%–30% smaller than other Cas13 subtypes, facilitating its flexible packaging and intracellular delivery.^69^ There is very limited information for Cas13c system.

Unlike Cas9, Cas13 proteins do not require PAM sequences to identify their targets, but they do have a protospacer flanking site (PFS) structure dependency, that is, that the base before the original spacer sequence should be A, C, or U.^70^, ^71^, ^72^ Notably, in eukaryotic and prokaryotic cells, Cas13 is activated after recognition and cleavage of RNA, but also has “collateral shearing” RNase activity, which can shear adjacent single‐stranded RNA and cause cell dormancy or programmed cell death.^70^, ^73^ Simultaneously, dCas13 was generated by inactivating the catalytically active domain of Cas13. A stronger inhibitory effect was observed with CasRx, an engineered variant of Cas13d, exerting a lower and virtually absent off‐target effect compared to CRISPRi (i.e., dCas9) as well as short hairpin RNA (shRNA) knockdown.^74^ In addition, inducible CRISPR artificial shearing factors (iCASFx) and multiplexing with orthogonal dCas13s were engineered, and their therapeutic potential was demonstrated in spinal muscular atrophy (SMA) fibroblasts.^75^ Altogether, the nonspecific RNase activity of Cas13 provides powerful tools for applications in nucleic acid detection, gene regulation, RNA imaging, and cancer therapy.^76^ Particularly, Cas13 has great potential for cancer therapy by targeted manipulation of key cancer‐associated RNA molecules (including mRNA and ncRNAs).^77^, ^78^

## 4 COMBINED APPLICATIONS OF CRISPR WITH SYNTHETIC BIOLOGY IN CANCER

Besides its naturally occurring form, the CRISPR–Cas system can also be modified by other means. Synthetic biology is a combination of engineering and biology disciplines that uses engineering design concepts to modify or create artificial living systems, often in a way that rewires naturally occurring biological circuits (either genes or proteins) to achieve the desired logical forms of cellular control.^79^ In the last decade, synthetic biology has begun to develop rapidly, with a series of pioneering milestones such as the smallest artificial synthetic cell (named JCVI‐Syn3.0),^80^ and artificial cells that can grow and divide normally.^81^ Gradually, we could “read,” “write,” and “compile” the genome, and have the ability to design and synthesize life. Today, biological design by applying engineering principles such as standardization,^82^ modularity,^83^ digital logic,^84^ and mathematically predictable behavior^85^ has become central to synthetic biology.

Notably, synthetic biology is developing rapidly in the field of medicine and is bound to have a dramatic impact on the medical field.^86^ For example, Williams et al.^87^ constructed a diverse library of multi‐receptor cell–cell recognition circuits by using synthetic Notch receptors to allow engineered T cells to achieve precise recognition of extra‐ and intracellular antigens through AND/OR/NOT gates. This approach opened up new avenues for precise identification of cells and was expected to be used to regulate the expression of target genes and kill target cells. Importantly, the application of combinatorial optimization strategies in synthetic biology, such as DNA barcoding tools and high‐throughput screening biosensors, allows us to achieve a best outcome.^88^ In parallel, synthetic biology is showing great fascination in the medical field as new developments in biotechnology are expanded into its toolkit.

The construction of genetic engineering at the cell level by CRISPR‐based technologies has greatly contributed to our understanding and controlling mammalian biological systems. The lack of effective gene activators makes it difficult to regulate gene expression efficiently. Dong et al.^89^ developed a synthetic bacterial transcriptional activator in *E*. *coli* by linking the activation domain to a programmable CRISPR–Cas DNA binding domain. The entire gene expression program can be turned on by inducing expression of the CRISPR–Cas systems. Thus, manipulating CRISPR technologies with engineering principles enables an efficient regulation of gene expression and provides the basis for engineering synthetic bacterial cellular devices. Meanwhile, the successful application of CRISPR systems armed with synthetic biology underscored their robust potentials in medicine.

### 4.1 Genetic circuits in cancer

**FIGURE 3:**
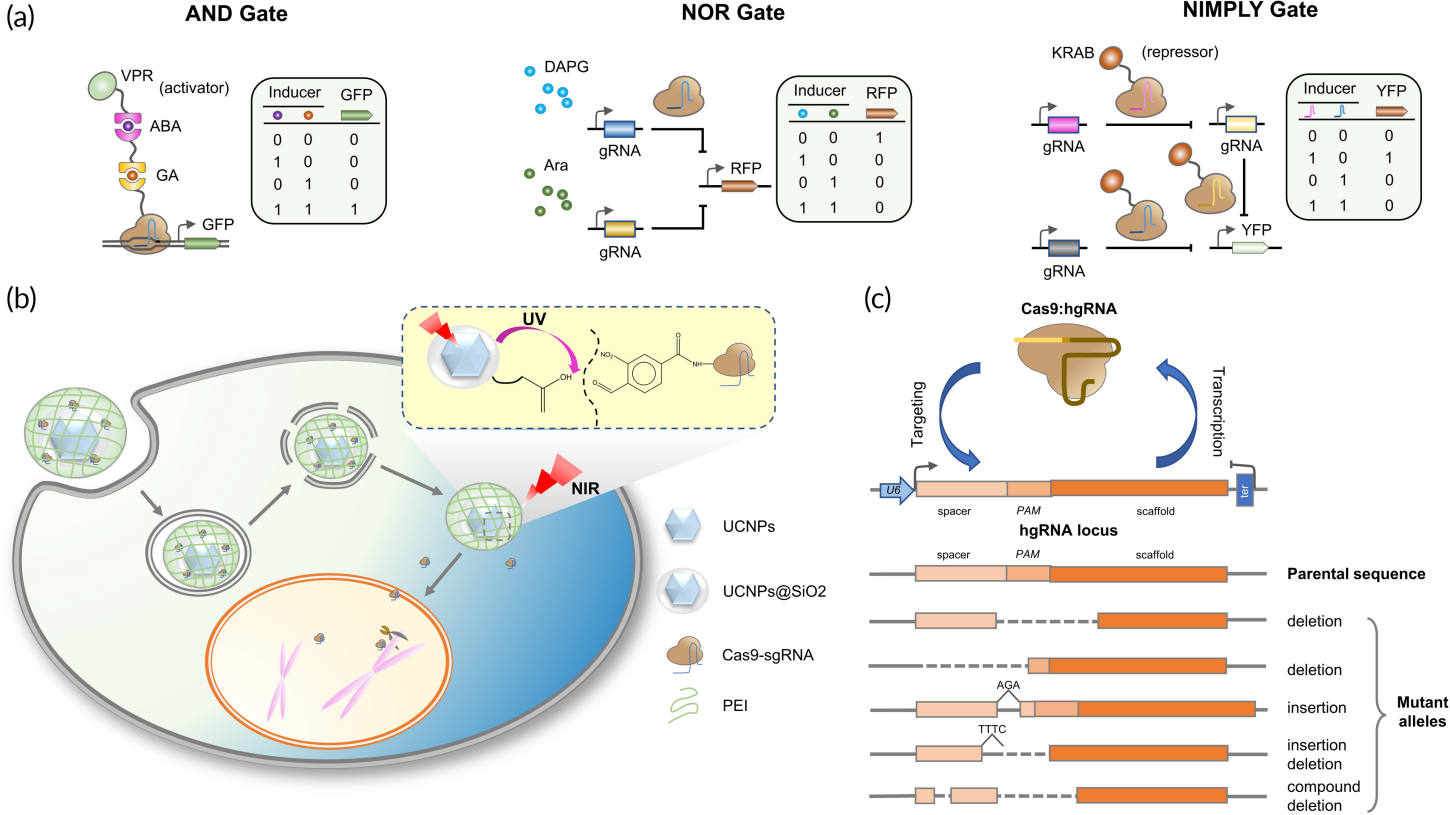
Applications of CRISPR–Cas systems with synthetic biology. (a) Construction of logic gates using CRISPR. Logic gates such as AND, NOR, and NIMPLY gates (shown here) allow greater specificity and range of antigens for targeting by engineered cells.^90^ (b) NIR‐triggered delivery of Cas9‐sgRNA to the nucleus of the cell for gene editing. The vector attaches to the cell membrane and enters the cell by endocytosis. Eventually, Cas9‐sgRNA is released from the pellet and steps into the nucleus. After the target DNA sites are identified, DNA double‐strand breaks are initiated for genome editing. (c) In the homing CRISPR system, the Cas9:hgRNA complex cleaves the hgRNA locus. When the NHEJ repair system repairs the cleavage, mutations can be introduced in the hgRNA locus. These mutations can effectively act as barcodes [Correction added on February 13, 2023, after first online publication: Reference 90 citation has been included figure 3(a) caption]

The robust and precise switching on and off one or more genes of interest is essential for many biological circuits as well as for industrial applications. Synthetic biology aims at designing modular genetic circuits. At present, CRISPR‐based genetic circuits include logic gates, cascades, bistable switches, and temporal and spatial pattern generators, among which logic gates are the most widely explored^90^ (Figure 3a).

Initially, CRISPR systems were used for the design and application of individual logic gates. The dCas9‐Mxi1‐based NOR gate designed in Saccharomyces cerevisiae enables direct conversion of gRNA inputs into gRNA outputs, allowing the gates to be “wired” together. It implements arbitrary internal logic for a variety of synthetic cellular decision‐making systems and shows minimal leak transcriptionally and digital responses, forming the basis for large, synthetic, cellular decision‐making systems.^91^ By using scaffold RNA (scRNA), Hofmann et al.^92^ designed a logic AND gate dependent on two independent switchable parts, namely dCas9 and MCP‐VP64 expression, controlled by galactose or β‐estradiol addition, respectively. A mathematical model and single‐cell analysis showed that the AND gate could only be activated in presence of both galactose and β‐estradiol, which in turn induced transcriptional activation and sensitive tunability. The scRNA CRISPR‐dCas9 platform provides an expression control system that can bind different components to an AND gate to achieve expression of multiple target genes with a tunable fold activation.

The complex intracellular signaling limits the application of individual logic gates, and the combined design of multiple logic gates demonstrates a more refined intracellular response. Peng et al.^93^ first used CRISPR–Cas12a system to successfully constructed three 2‐input elementary AND, OR, and INHIBIT logic gates. These logic gates are capable of normal operation and can be used for rapid and sensitive detection of external pathogenic bacteria such as *Staphylococcus aureus*. On the other hand, to detect diverse types of disease‐relevant cues, Kempton et al.^94^ developed a split Cas12a platform capable of spontaneously reassembling and demonstrated that it achieved construction of multi‐input, multi‐output logic circuits in mammalian cells. The platform was highly programmable and allowed expandable AND gates with two, three, and four inputs. The availability of anti‐CRISPR systems served as an OFF switch via incorporating NOT logic. In terms of output, the platform can generate activation and repression of target genes or permanent modification of genomic DNA. Thus, split dCas12a can achieve specific activation of the anti‐tumoral programs in cancer cells by coupling to multiple cancer‐relevant inputs.

Single nucleotide mutations (SNMs) are extremely important in cancer, and multiple SNMs further complicate the pathogenesis of cancer.^95^ Therefore, performing multi‐SNMs detection and analysis facilitates cancer screening. The logic gate circuits based on CRISPR systems enable the detection of multiple SNMs. Long ssDNA fragments with guanine‐rich sequences were known to be generated after CRISPR–Cas9 system shear. The G‐quadruplex/ThT (G4/ThT) complex, as fluorescent probe, can be formed by introducing thiaxanthin T (ThT) into the G‐quadruplex. Complicate genetic locus could be distinguished by designing an AND logic gate system. The operation was set as the recognition of two mutation sites by sgRNAs as input signals and the fluorescence signals as output signals. Specifically, the input case was defined as (1,1) when both sgRNA1 and sgRNA2 existed. Subsequently, the G4/ThT complex was formed after Cas9n/sgRNA complex recognized the two cleavage sites. The fluorescent signal was activated and the process was treated as “ON”. The input cases (1, 0 or 0, 1 or 0,0) generated insufficient fluorescent signal, and the output was considered as “OFF.”^96^ Furthermore, the human telomerase reverse transcriptase (hTERT) promoter is considered to be a cancer‐specific promoter,^97^ while the human uroplakin II gene (hUP II) promoter is a bladder‐specific promoter.^98^ Liu et al.^99^ designed an AND gate based on CRISPR–Cas9 system by combining hTERT and hUP II. The hTERT and hUP II promoters served as input signals to drive transcription of Cas9 mRNA and sgRNA targeting LacI, respectively. The luciferase reporter was used as the output gene. The results showed that the circuit could detect bladder cancer cells specifically and effectively inhibit the growth of cancer cells by regulating related genes. Aiming to further improve the efficiency of cancer diagnosis so as to facilitate clinical translation, Liu et al.^100^ improved and optimized the previous logic circuit to develop AND gate minigene circuits based on CRISPReader. The results showed that the minigene circuit had higher cancer recognition and intervention ability in vitro and stronger anti‐cancer effects in vivo than the conventional gene circuit. It can be anticipated that the minigene circuit will take the advantages of gene circuits in synthetic biology to further optimize precision medicine research in malignant tumors and other diseases. With the advantages of high programmability, modularity, and orthogonality, CRISPR‐based genetic circuits will enable us to construct more complicated and sophisticated synthetic circuits, making them the more powerful therapeutics for cancer therapy and other medical treatments.

### 4.2 Optogenetic devices in cancer

CRISPR systems‐mediated gene editing has been well‐established as a powerful tool for in vitro and in vivo gene regulation, but has not yet been able to achieve such regulation in spatial and temporal manners. Optogenetics offers an unprecedented ability to achieve precise spatial and temporal control of cellular activity using light of appropriate intensity and wavelength as a trigger signal.^101^, ^102^ Hence, incorporating optogenetics into CRISPR‐based gene regulation system could circumvent such a limitation.

Polstein et al.^103^ engineered a CRISPR–Cas9 effector (LACE) system that induced endogenous gene transcription under blue light by fusing light‐inducible heterodimerizing proteins CRY2 and CIB1 to a transactivation domain and the catalytically inactive dCas9, respectively. The LACE system can be readily directed to DNA sequences and reversibly regulate endogenous genes by light, suggesting that optogenetic systems offer a greater flexibility and simplicity in targeting endogenous genes. Meanwhile, Niopek et al.^35^ described optogenetic anti‐CRISPR variants named CASANOVA, which include AcrIIA4 (a potent Streptococcus pyogenes Cas9 inhibitor) and the LOV2 photosensor from Avena sativa. These artificial Acr proteins enable light‐mediated genome and epigenome editing via a precise external blue light stimulus. By co‐expressing CASANOVA and sgRNAs targeting different genomic loci in HEK293T cells, insertion/deletion (indel) mutations at all target loci were strongly light‐dependent. The potential of CASANOVA for Cas9 DNA targeting kinetics in living cells was further confirmed by irradiating cells with a 488 nm laser beam. Again, Niopek et al. turned their attention to the type II‐C Cas9 from *Neisseria meningitidis* (NmeCas9), which is much smaller than SpyCas9 with only 1081 amino acids. It exhibits high target specificity, probably related to its longer target recognition sequence (~24 nucleotides) and a longer PAM sequence (N4GATT for NmeCas9 versus NGG for SpyCas9).^104^ A light‐dependent anti‐CRISPR protein named CASANOVA‐C3 was successfully developed for conditional activation of NmeCas9. Namely, NmeCas9 activity could be potently blocked in the dark, but activated under blue light, allowing reversible genome editing at various endogenous loci which could be precisely monitored and controlled using light signals.^105^

Accordingly, Qi et al.^106^ designed a blue light photosensor that can effectively induce the expression of Cas13a protein and precisely control the expression of MALAT1 which was confirmed to be an oncogene in human bladder cancer. The results showed that the MALAT1 expression level in bladder carcinoma cell lines (5637 and T24) was significantly suppressed and the malignant phenotype of bladder cancer cells was alleviated under blue light irradiation. This study provides a proof of concept for novel cancer treatment using the newly created CRISPR‐based gene editing system.

Due to their poor penetration and possible cytotoxicity, neither UV light nor blue light can be widely used for in vivo research applications and clinical translation.^107^, ^108^ Considering that red light could penetrate deep tissue more than 5 mm beneath skin surface,^107^ the development of red light‐activating CRISPR systems using nanotechnology can further expand the application of optogenetic devices in cancer. A CRISPR–Cas9 nanosystem was generated using the cationic polymer‐coated Au nanorods (APC) and Cas9 plasmids driven by the heat‐inducible HSP70 promoter. APC not only serves as a vector for plasmid delivery, but also functions as an intracellular NIR light photothermal converter to induce Cas9 expression. Data from the in vitro and in vivo studies confirmed good sensitivity and inducibility of this new system, with minimal off‐target effects.^109^ Furthermore, Pan et al.^110^ reported an upconversion nanoparticles (UCNPs)‐based near‐infrared light (NIR)‐responsive CRISPR–Cas9 vector named UCNPs‐Cas9@PEI. UCNPs could convert NIR to localized UV light for cleavage of photosensitive molecules. NIR light stimulation induced the on‐demand release of CRISPR–Cas9, which enabled controlled gene editing. Serine/threonine‐protein kinase PLK‐1, also known as polo‐like kinase 1 (PLK‐1), has been implicated in many different cancers, therefore targeting PLK‐1 represents one of the novel therapeutics for cancer treatment.^111^, ^112^ Thus, by designing sgRNAs targeting PLK‐1, the anti‐tumor activity of this system can be explored. Indeed, the expression level of PLK‐1 in A549 cells was significantly inhibited with sgRNAs targeting PLK‐1. Meanwhile, tumor growth was also significantly arrested in xenograft nude mice model bearing A549 cells. This study provides clear evidence to confirm that the release of CRISPR–Cas9 with anti‐tumor potential from UCNPs‐Cas9@PEI can be precisely controlled by NIR for cancer therapy (Figure 3b). These nanoCRISPR systems with optical properties provide a programmable genome editing strategy capable of treating deep tissue tumors. Apart from NIR, far‐infrared light (FRL) has also been explored for cancer treatment. Yu et al.^113^ designed an FRL‐activated split‐Cas9 (FAST) system, with two components, namely N‐terminal Cas9 fragment [Cas9(N)] linking with the Coh2 domain and C‐terminal Cas9 fragment [Cas9(C)] linking with the DocS domain. Coh2 and DocS are two *C. thermocellum* proteins that interact with high affinity. Under FRL illumination (730 nm), the FAST system was successfully assembled in HEK‐293 cells and activated for target genome editing. In a mouse xenograft tumor model, FRL‐triggered PLK‐1 oncogene editing was successfully achieved using FAST system. The system thus extended the spectrum of light energies in optogenetic toolbox, allowing for the noninvasive induction of gene editing activity in cells located in deep tissues. Taken together, the optogenetic control of CRISPR systems greatly improves our ability to achieve highly accurate genomic perturbations spatiotemporally in living cells for cancer treatments.

### 4.3 Cellular barcoding for cancer therapy

Cellular barcoding involves individual cells being tagged with unique nucleic acid sequences so that they can be tracked through space and time. At present, cellular barcoding has been widely adopted for fate mapping, lineage tracing, and high‐throughput screening, and has greatly contributed to the understanding of developmental biology and gene function.^114^ Alejo Rodriguez Fraticelli et al.^115^ have traced and characterized the family tree of individual blood cells at the time of their formation in their natural environment by tagging bone marrow cells from mice with barcodes. Their results confirmed that the megakaryocyte lineage was the predominant native fate of long‐term hematopoietic stem cells. Meanwhile, cellular barcoding can also be used for cancer surveillance. Researchers used cellular barcoding of breast cancer xenografts (PDXs) to track the engraftment and growth characteristics of individual cells in early passaged PDX tumors. The authors observed the proliferation characteristics of tumor cells and found that chemotherapy only temporarily reduced the number of harmful cells and did not permanently eliminate them.^116^ Such findings significantly improved our understanding of the mechanisms of breast cancer metastasis and drug resistance.

Cellular barcoding technology has been introduced into CRISPR systems to obtain diverse editing patterns and informative loci. Cas9 was known to create insertions or deletions in gene sequences in the absence of a homologous repair template, and these insertions or deletions (genetic “scars”) constituted heritable cellular barcodes that can be read out by scRNA‐seq for genealogical analysis. Spanjaard et al.^117^ proposed a strategy that allowed simultaneous lineage tracing and transcriptome analysis in thousands of single cells, known as LINNAEUS (lineage tracing by nuclease‐activated editing of ubiquitous sequences). Injection of Cas9 and sgRNA into single‐cell stage embryos enabled the labeling of genetic scar cells at early developmental stages and allowed for tracing these cells in distinct organ developmental lineages in zebrafish larvae as well as adult fish. Such a strategy provides a new approach to study cancer single‐cell analysis and metastasis. In addition, new strategy with constantly self‐editing DNA barcodes in living cells has been proposed. Cas9 proteins can randomly mutate the sequence of guide RNA in cells, generating homing guide RNA (hgRNA). By performing confusion analysis, the parentage between different cell populations could be determined. When Cas9 was activated, the hgRNA sequence drifted randomly, acting as an evolutionary barcode. Indeed, multiple studies reported that this homing CRISPR–Cas9 system could be used as a genetic barcode, allowing for the controlled sequence diversification, deep lineage tracing, and molecular recording in cancers^118^, ^119^ (Figure 3c). In recent years, a whole‐organism lineage tracing has been developed, named genome editing of synthetic target arrays for lineage tracing (GESTALT), which can be used to map large‐scale cell lineage in multicellular systems.^120^ However, the disadvantages of being limited to early development and the inability to identify cell types restricts its application.^120^ Raj et al.^121^, ^122^ combined single‐cell RNA sequencing of identifiable cell types with GESTALT to generate scGESTALT, which allowed barcodes to be edited at multiple time points. Furthermore, the CRISPR array repair lineage tracing (CARLIN) system was established to simultaneously interrogate the lineage and transcriptomic information of single cells in vivo. Specifically, a universal mouse model was designed by using CARLIN, capable of generating up to 44,000 transcribed barcodes in an inducible manner at any time points during development or adulthood. The CARLIN system is therefore uniquely suited to the study of stem cell cloning dynamics.^123^ The abovementioned novel properties make CRISPR barcodes useful for a wide range of applications, such as dissecting cancer biology.

Notably, by establishing MDA‐MB‐231 and AT‐3 cell models with barcoding system and corresponding in vivo mouse models Zhang et al.^124^ confirmed the existence of re‐metastasis from bone metastases to other organs during spontaneous metastasis of mammary gland carcinoma in mice. Moreover, multiple combinations of genetic mutations have been implicated in cancer progression and interactions between different mutations are currently unknown. Rogers et al.^125^ proposed a parallel approach of tumor barcoding and high‐throughput barcode sequencing (Tuba‐seq) to quantify the impact of many different tumor suppressor gene alterations. By using CRISPR–Cas9‐mediated tumor suppressor inactivation, tumor barcoding, and deep sequencing of DNA barcodes, Tuba‐seq allows us to unravel novel insights into mutant gene interactions in genetically engineered mouse cancer models, and enables us to precisely track every single cell in tumors and significantly accelerate drug development for cancer.

DNA barcoding can label enormous numbers of cells, but can only provide volumetric resolution and does not yield high‐precision phenotypic and somatic cell resolution.^126^ To address these issues, a barcoding system that operated at the protein level has been reported. By synthesizing modules encoding linear epitope triplets, more than 100 unique protein barcodes (Pro‐Codes) can be generated, with each Pro‐Code can be paired with a different CRISPR, allowing for simultaneous analysis of multiple cancer phenotypic markers. Employing the Pro‐Code/CRISPR screening system, the authors reported that antigen‐dependent immune editing of cancer cells were controlled by two interferon‐stimulated genes, the immunoproteasome component Psmb8 and a chaperone Rtp4, and identified Socs1 as a negative regulator of Pd‐l1.^127^ It is thus clear that Pro‐Code/CRISPR enables phenotypic analysis of a large number of cancer genes with high precision cellular resolution, contributing to the genetic annotation of various cancer.^127^ Overall, combining cellular barcoding technology with CRISPR systems has dramatically enriched our understanding of cancers at both the genetic and molecular levels.

### 4.4 Genome‐wide or CRISPR screens for cancers

Forward genetic screens with genome‐wide CRISPR libraries are powerful tools for resolving cellular circuits and signaling pathways. Also, pooled CRISPR screens are widely used as a method to identify genes involved in biological mechanisms such as cell proliferation, drug resistance, and viral infection. For example, mutations in the MEN1 gene are known to induce tumorigenesis. Using a CRISPR–Cas9 screening system Ma et al.^128^ identified dihydroorotic dehydrogenase (DHODH) as a synthetic lethal gene partner of MEN1 in MEN1‐mutated tumor cells. DHODH is primarily found in the inner mitochondrial membrane and is a key enzyme for the de novo synthesis of pyrimidine nucleotides. In malignantly proliferating cells, the cells are dependent on the de novo synthesis pathway to produce sufficient pyrimidine nucleotides.^129^ Therefore, inhibition of DHODH in malignant cells can induce cell death. In MEN1 mutation‐associated tumors, DHODH expression was enhanced due to the absence of MEN1 protein, leading to massive proliferation of tumor cells. Excitingly, leflunomide is a potent DHODH inhibitor.^130^ By constructing a mouse xenograft model, the researchers administered leflunomide to MEN1 mutation‐associated tumors and showed a significant reduction in tumor size.^128^ Meanwhile, through forward genetic screens, CRISPR technologies allow high‐resolution detection of genetic traits in cancer cells. A genome‐wide CRISPR–Cas9 screen of pancreatic ductal adenocarcinoma (PDAC) cells with RNF43 mutations identified the Wnt receptor frizzed‐5 (FZD5) as the only FZD receptor encoded in the human genome, suggesting that antibodies to FZD5 could be used to inhibit PDAC cell proliferation.^131^ A genome‐wide CRISPR screen can also be used to reveal the genetic circuits by which cancer cells evade the host immune system. Lawson et al.^132^ performed a genome‐wide CRISPR screen on mouse cancer cell lines cultured in the presence of cytotoxic T lymphocytes (CTLs). The results showed that 182 core genes were involved in regulating the sensitivity or resistance of cancer cells to CTL‐mediated toxicity. These studies provide clear evidence to support that CRIPSR‐based genome‐wide genetic screens can be used to identify and validate cell surface targets and core genes for developing antibody treatments and gene therapy for cancers.

**FIGURE 4:**
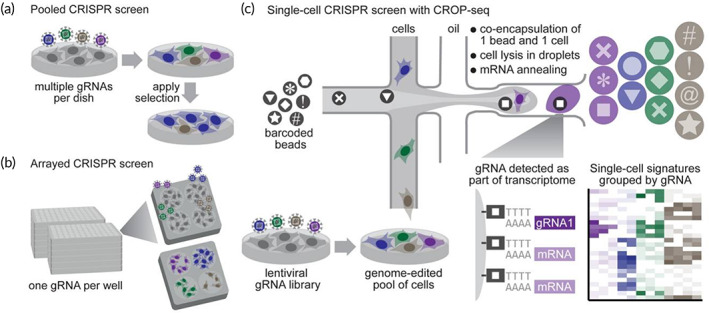
The main methods of CRISPR‐based screens. (a) Pooled CRISPR screens detect changes in gRNA abundance among bulk populations of cells. However, they do not support complex molecular readouts such as transcriptome profiling. (b) Arrayed CRISPR screens support complex readouts such as transcriptome profiling, but with a much lower throughput. (c) A CRISPR droplet sequencing (CROP‐seq) that combines the key advantages of pooled screens and array screens has been developed^135^

Large‐scale screening of complex cellular phenotypes is a shortcoming of the CRISPR screens, and single‐cell RNA sequencing offers a solution to this problem. By combining the advantages of single‐cell RNA sequencing and pooled CRISPR screens, Dixit et al.^133^ constructed Perturb‐Seq which allowed the functions of different genes in different cell types in an organism to be studied simultaneously. It has been shown that Perturb‐seq is able to accurately and simultaneously determine the cellular states with distinct gene responses among 200,000 immune cells and in proliferating cell lines. Meanwhile, CRISP‐seq was developed to study the crosstalks and redundancies in complex genetic circuits and massive cellular heterogeneity in multicellular organisms. Specifically, CRISP‐seq has shown clear potential in coding genes, knocking out genes, disrupting genetic elements such as noncoding RNA, promoters, and enhancers, as well as inducing specific gene expression.^134^ Moreover, Datlinger et al.^135^ developed a CRISPR droplet sequencing method (CROP‐seq) by combining key strengths of pooled and arrayed screens to enable pooled CRISPR screens with single‐cell transcriptome resolution, which will facilitate high‐throughput functional dissection of complex regulatory mechanisms and heterogeneous cell populations (Figure 4a–c). Furthermore, Wheeler et al.^136^ reported a method named as CRaft‐ID that combines pooled CRISPR–Cas9 screen with microraft array technology and high‐content imaging to screen image‐based phenotypes. CRaft‐TD extends the application of CRISPR screens to the throughput of image‐based genetic knockout studies, enabling us to study genetic modulators of subcellular and cellular phenotypes.

Moreover, improving the accuracy and robustness of pooled‐library CRISPR screens by capturing sgRNA integrations in individual organoids can significantly reduce required cell numbers for genome‐scale screening. Genetic dissection of the TGF‐β tumor suppressor pathway can be achieved by establishing a genome‐level CRISPR screen for human small intestine (hSI) organoids.^137^ Additionally, Michels et al.^138^ developed a platform for pooled CRISPR–Cas9 screen in human colon organoids to facilitate high‐throughput genetic testing and functional identification of tumor drivers. In a screened pan‐cancer tumor suppressor gene (TSG) library, TGFBR2 was shown as the most prevalent TSG. The platform for pooled CRISPR–Cas9 screen in human colon organoids permits unbiased detection of genes that confer positive selection in vitro and after xenotransplantation. The successful use of pooled CRISPR screens for human organoids, therefore, provides a powerful platform for identifying patient‐specific vulnerabilities and for genetically dissecting cancer mechanisms in physiologically relevant model systems.

## 5 CURRENT DELIVERY SYSTEMS OF CRISPR–Cas

**FIGURE 5:**
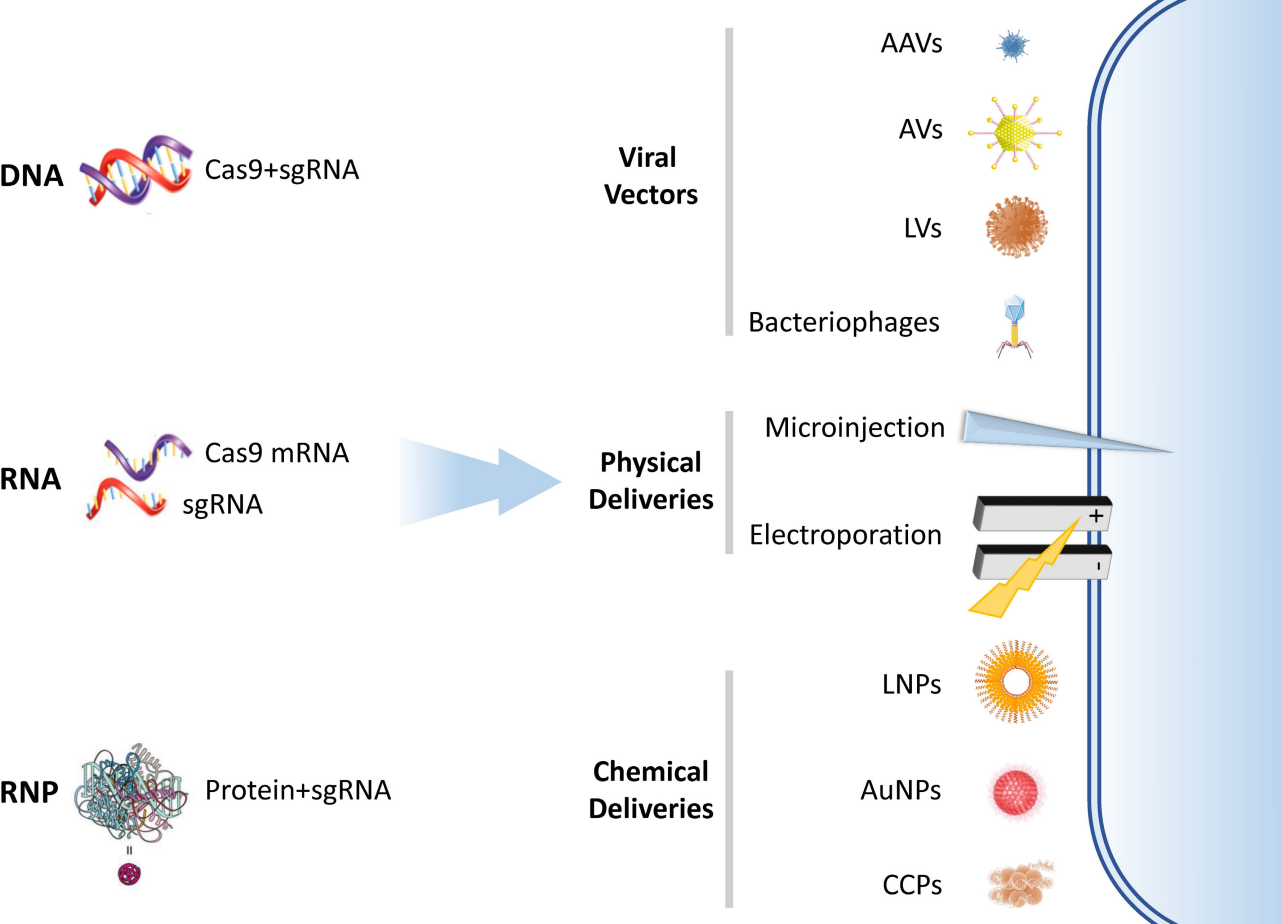
The current delivery forms and strategies of CRISPR–Cas9. CRISPR can be delivered as DNA, RNA, or ribonucleoprotein (RNP). Each form of delivery has advantages and disadvantages. Specific details can be found in Table 2. Regardless of delivery forms, the largest challenge lies in delivering the cargo across the membrane. A variety of viral, physical, and chemical methods have been derived to achieve successful delivery across the cell membrane

The multiple forms and derivatives of the CRISPR–Cas systems as discussed in above sections significantly enrich their applications in cancer at the genetic level. However, the commonly used vector delivery is an urgent issue for CRISPR–Cas systems to enter into human clinical trials. Currently, three main delivery forms are used for delivering CRISPR–Cas9 (Figure 5): (i) Delivering ribonucleoprotein (RNP, a complex of Cas9 protein and sgRNA)^139^, ^140^; (ii) Delivering mRNA and sgRNA components^141^; (iii) Delivering plasmid DNA encoding Cas9 and sgRNA, such as pX330, pX458, and pX459.^142^, ^143^ The advantages and disadvantages of these three approaches are shown in Table 2. The CRISPR–Cas systems for targeting DNA must enter the nucleus of the target cell in order to have a therapeutic effect. To achieve such a purpose, a number of delivery systems have been developed for the CRISPR–Cas9 system. Depending on whether viral transduction is used, CRISPR–Cas9 delivery strategies can be broadly classified as viral or nonviral approaches, the latter also includes a variety of physical and chemical delivery strategies (Table 3).

**TABLE 2:** CRISPR/Cas9 system delivery formats

Delivery formats	Advantages	Disadvantages	References
Protein RNP	Rapid; significant reduction in off‐target effects, toxicity, and immune response	Difficult to obtain large amounts of highly active Cas9 protein; large Cas9 protein size	^139^, ^140^
Cas9 mRNA and sgRNA	Faster; favorable for reducing off‐target effects	Poor mRNA stability; poor gene editing	^141^
Plasmid DNA	Simple; stable; avoiding multiple transfections	Low efficiency; higher off‐target effect; difficult translocation of plasmid DNA to the nucleus	^142^, ^143^

**TABLE 3:** Delivery strategies for various CRISPR–Cas formats

	Viral delivery				Physical delivery			Chemical delivery			Other emerging nonviral delivery			
Strategy	AAVs	AVs	LVs	Bacteriop‐hages	Microinjection	Electroporation	Hydrodynamic injection	Cationic vectors	CCPs	AuNPs	GO	BP	DNA nanostructure	EVs
Delivery formats	DNA	DNA	DNA	DNA	DNA, mRNA or protein	DNA, mRNA or protein	DNA, mRNA or protein	DNA, mRNA or protein	Protein	Protein	Protein	Protein	Protein	Protein
Delivery efficiency	++	++	+++	++	+	+++	+++	+	+	++	+++	+++	+++	++
Safety concern	+	++	+++	+	+	+	++	+	+	+	+	+	+	+
Cost	++	++	+	++	+++	+++	++	+	+	++	++	++	++	+
Technical requirement	++	+++	+	++	+++	+	+++	+	++	++	++	+++	+++	+
Major applications	in vivo	in vivo	in vitro and ex vivo	in vivo and in vivo	in vitro and ex vivo	in vitro and ex vivo	in vitro and ex vivo	in vitro and in vivo	in vitro and in vivo	in vitro and in vivo	in vitro and in vivo	in vitro and in vivo	in vitro and in vivo	in vitro, ex vivo and in vivo

Abbreviations: + denotes low; ++ denotes medium; +++ denotes high; AAV, adeno‐associated virus; AuNPs, gold nanoparticles; AV, adenovirus; BP, black phosphorus; CCPs, cell penetrating peptides; EVs, extracellular vesicles; GO, graphene oxide; LV, lentivirus.

### 5.1 Viral vector delivery

Over 70% of the gene therapy in clinical trials are using viral vector to deliver therapeutic genes, which is accomplished with two main mechanisms: infection and replication. During the infection phase, the virus can identify and enter a specific cell and the viral genome will be released into the nucleus (DNA) or cytoplasm (RNA) for replication.^144^ Gene therapy by genome editing is achieved by transporting a virus containing the delivery material (programmed genome editing nuclease) to a target cell. A large number of viral vectors have been developed, mainly adeno‐associated viruses (AAVs), adenoviruses (AVs), lentiviruses (LVs), and bacteriophages.^145^, ^146^

#### 5.1.1 Adeno‐associated viral vector‐mediated delivery

AAV vectors are a common viral vector used for gene therapy. Compared to other viral vectors, AAV vectors are widely used for delivery of CRISPR–Cas9 system due to their low immunogenicity, long‐lasting transgene expression, mild immune response, high infection efficiency, and general safety.^144^, ^147^ However, the low packaging capacity of the AAV vectors (<4.7 kb) has limitations for delivery of large size Cas proteins.^148^, ^149^ To address this limitation, two methods of delivery are now commonly used. The first one is looking for and using smaller‐sized Cas9 proteins. The most commonly used SpCas9 (~4.2 kb) is challenging to deliver, while the smaller derivatives including SaCas9 (~3.2 kb), St1Cas9 (~3.4 kb), and NmeCas9 (~3.2 kb) are feasible for AAV delivery.^150^ However, the smaller Cas9 proteins are restricted by the availability of suitable PAM sequences. Second, a dual‐AAV vector system was designed, with one AAV vector delivering spCas9 protein and the other delivering gRNA.^151^ Notably, the efficiency of delivery is diminished due to the reduced probability of two AAV vectors delivering to the same cell. Therefore, AAV vectors for delivery of CRISPR–Cas9 system need to be further optimized for greater clinical applicability. In this aspect, Richards et al.^152^ engineered a dual‐AAV vector to deliver CRISPR/Cas9 machinery and co‐administered a nonhomologous end‐joining inhibitor vanillin to promote homology‐directed repairs, and found that the Pah^*enu2*^ allele was permanently corrected in the hepatocytes of mice with phenylketonuria. A triple AAV carrier has also been engineered to accommodate the larger size of the load and improve the delivery efficiency.^153^ Therefore, clinical trials with AAV‐delivered CRISPR/Cas9‐based gene editing are expected to be starting shortly as the AAV delivery system becomes increasingly refined.

#### 5.1.2 Adenovirus‐mediated delivery

AVs are a nonenveloped double‐stranded DNA virus that could transduce both dividing and nondividing cells. In addition, they have no endogenous integration mechanism and do not integrate into the host cell genome.^154^ AVs are widely used in clinical trials for gene therapy because of their good biological properties, genetic stability, and high gene transfer efficiency.^155^ Kato et al.^156^ developed an AV vector that could edit the X gene in HBV cell lines and showed that the AV vector successfully blocked the HBV‐X gene in heterogeneous patients. However, AVs tend to trigger innate immune responses within host cells, leading to tissue inflammation. This, coupled with the laborious production of AVs, further limits the efficiency of clinical application.^157^, ^158^

#### 5.1.3 Lentiviral vector‐mediated delivery

LVs derived from HIV‐1 viruses have tropism and larger cargo capacity (~8 kb), which allow Cas9 and gRNA to be loaded into an LV vector simultaneously.^159^ LVs have the ability to transduce nondividing cells efficiently compared to other viral vectors, making them an instrumental tool for gene therapies in somatic and germline cells.^160^ Since all viral genes are deleted, the LV vector does not activate the immune system.^161^ Furthermore, the production of LVs is simpler than that of AAVs and AVs.^162^ Taking these advantages, Holmgaard et al.^163^ successfully delivered SpCas9 protein and sgRNA via LV vector to achieve Vegfa genome editing in the mouse retina. Similar to AVV vectors, dual‐LV vector systems were developed for efficient, time‐controlled deletion of MCL‐1 (a gene controls apoptosis, making tumor cells resistant to chemotherapeutic agents) in vitro and in vivo.^164^ However, as a retrovirus, the host genome integration of LVs leads to unwanted off‐target insertional mutagenesis that may result in the inactivation of tumor suppressor genes or the activation of proto‐oncogenes and thus induce tumorigenesis.^165^, ^166^ Clinical trials of LV vector‐delivered CRISPR–Cas systems are being shelved due to potential risks of tumorigenesis.

#### 5.1.4 Bacteriophage‐mediated delivery

Bacteriophages are a group of viruses that infect bacteria or archaea and are often used against multidrug‐resistant bacteria. However, the specific susceptivity of bacteria has limited the development of bacteriophage therapy. Fortunately, the combination of bacteriophages and the CRISPR–Cas system has changed this dilemma. Bikard et al.^167^ designed a plasmid containing the cas9 gene and its gRNA sequence, and delivered it to *S*. *aureus* using bacteriophage. The results showed that the delivery system was effective in killing *S*. *aureus* on the skin surface of mice. Subsequently, various CRISPR–Cas systems using bacteriophage delivery have been widely used for controlling bacterial infections. Selle et al.^168^ used bacteriophages to successfully deliver CRISPR–Cas3 and significantly inhibit *C*. *difficile* infection. Using a similar strategy, Liu et al.^169^ delivered the bacteriophage‐mediated CRISPR/Cas9 to drug‐resistant bacteria and showed a sustained and efficient eradication of drug‐resistant bacteria. Despite the huge success in controlling bacterial infection, there is little evidence to show the application of bacteriophage‐mediated CRISPR–Cas delivery and gene editing in cancer treatment.

### 5.2 Nonviral delivery

Several nonviral vectors have been developed and successfully applied for CRISPR–Cas delivery (either DNA, mRNA, or protein), such as physical methods to disrupt the cellular barriers, chemical modifications to improve cargo transport to avoid the barriers, and physical encapsulation of the cargo in the vector molecule.^170^ The main advantages of nonviral vectors are their ability to accommodate large size delivery, ease of generation, good controllability, and safety.

#### 5.2.1 Physical delivery

Delivery of CRISPR–Cas is achieved by exposing cells to mild physical conditions that temporarily disrupt the physical barriers that prevent the cargo from reaching its intended destination. Microinjection is a physical method of injecting Cas9 and sgRNAs directly into cells using a microscope and a microinjection needle.^171^ This method has been used for DNA, RNA, or RNP delivery of Cas9, as well as for direct delivery of gRNAs.^171^, ^172^, ^173^ However, the method is heavily relied on well‐established experimental facilities and delicate handling to avoid permanent damage to the membrane, and is therefore commonly used for in vitro cell experiments. Electroporation is the temporary disturbance of the lipid bilayer of the plasma membrane by an electric field, thereby enhancing the permeability of the cell membrane.^174^ The method has been successful in delivering DNA, RNA, and even RNPs in vitro.^139^, ^175^, ^176^ However, electroporation‐mediated gene editing is costly and requires specific induction conditions to be set for different cell types. Importantly, high level of cell death caused by electroporation also limits its clinical application.^177^ Both physical methods have been widely used in vitro, but only a few strategies have been used for in vivo delivery of the CRISPR–Cas system, including hydrodynamic injection.^178^ Hydrodynamic injection enables delivery of CRISPR–Cas by creating temporary pores in the cell membrane at high pressure for a short period of time.^179^ For example, using hydrodynamic injection of pX330‐PTEN plasmid, Yu et al.^180^ effectively decreased the expression level of PTEN in rat liver and successfully induced lipid deposition and nonalcoholic fatty liver. Currently, the method can only be applied to small animal models due to the large starting injection volume.^179^ Therefore, it is not currently suitable for human application.

A number of new physical methods are being developed for CRISPR–Cas component delivery. Sessions et al.^181^ proposed a scheme, named lance array nanoinjection (LAN) that produced transient pores (1–2.5 μm diameter) in the target cell membrane. The results showed successful transfer of the CRISPR/Cas9 plasmid into the GFP+/FRT HeLa cell line and the eventual modification of the EGFP gene. Importantly, LAN did not cause massive cell death, unlike with electroporation. Additionally, ultrasonically driven nanomotors have exhibited direct and rapid cell membrane permeation and have been applied to the intracellular delivery of ncRNA.^182^ Wang et al.^183^ loaded Cas9/sgRNA complex onto the surface of nanomotors by disulfide bond modification. The obtained nanomotors could directly penetrate the cytoplasm of GFP‐expressing mouse melanoma B16F10 cells after ultrasound treatment. The results showed that the GFP gene was efficiently knocked out. Taken together, these newly developed physical methods offer potential alternative strategies for delivery of the CRISPR–Cas systems to tumor cells.

#### 5.2.2 Chemical delivery

Physical methods are readily available in the laboratory setting, but less practicable for clinical application. Chemical deliveries use complementary components to assist Cas9 in bypassing cellular barriers and protect it from degradation. There are two main forms of chemical delivery: encapsulation of cargos in another chemical entity, or direct chemical modification of cargos, which mainly includes cationic vectors [such as lipid nanoparticles (LNP), cell penetrating peptides (CPPs), and inorganic metal nanoparticles (NPs)].^184^, ^185^, ^186^

As both nucleic acids and cell membranes are negatively charged, the repulsive forces between them prevent nucleic acids from entering the cell. Cationic modifications can, on the one hand, mediate binding to the nucleic acid through electrostatic interactions and protect the nucleic acid from destruction by nucleases. On the other hand, it assists the nucleic acid to cross the cell membrane and complete the delivery of the cargo.^187^, ^188^ Currently, there are two main categories of cationic‐based vectors: cationic lipid‐based vectors (LNP) and cationic polymer‐based vectors. LNP, in which negatively charged nucleic acids can be encapsulated, has been widely used for the delivery of ncRNAs and the like. This method is less efficient in delivery, but is safe, simple, and inexpensive.^31^ LNP has also been used to deliver CRISPR–Cas9 system. For example, Zuris et al.^189^ successfully transfected gRNA and Cas9 RNP complexes into a HeLa reporter cell line via cationic liposomes, achieving an 80% gene modification rate. Moreover, sgRNA targeting the DPP‐4 gene was designed to form a RNP with Cas9 protein and encapsulated with lecithin cationic liposomes. The results showed that the nano‐preparation resulted in the destruction of the DPP‐4 gene in type 2 diabetic mice.^190^ Recently, encapsulation of focal adhesion kinase (FAK) siRNA, Cas9 mRNA, and sgRNA (siFAK + CRISPR‐LNPs) in LNP achieved a 10‐fold enhancement of gene editing in tumor spheroids. By further constructing siFAK + CRISPR‐PD‐L1‐LNPs, the expression of PD‐L1 was effectively blocked, and the tumor growth and metastasis of the four cancer mouse models were also significantly inhibited.^191^ LNP can also be combined with physical methods. Yin et al.^192^ combined the LNP delivering Cas9 mRNA with AAVs encoding a sgRNA and a repair template to form a recombinant template after hydrodynamic tail vein injection, increasing repair efficiency from 0.4% to 6%. However, these vectors are currently mainly used for gene editing at the cellular level since they do not contain reactive functional groups, with poor tissue and cellular targeting ability and low in vivo stability.

Cationic polymer‐based vectors offer a wider range of options and more flexible structural design than cationic lipid vectors. Polyethyleneimine (PEI) is a commonly used polymer for CRISPR–Cas9 delivery. Ryu et al.^193^ found that branched PEI 25 kDa (BPEI‐25K) was effective in delivering plasmids expressing gRNA and Cas9 into Neuro2a cells for successful gene editing of Slc26a4 locus. Furthermore, the joint design of multiple polymers further expands the tool box. Luo et al.^194^ encapsulated Cas9 expression plasmids (pM458 and pM330) in cationic lipid‐assisted PEG‐b‐PLGA nanoparticles (CLAN) to achieve gene editing in macrophages in vivo and in vitro, respectively. By designing sgRNAs targeting Ntn1 gene, CLAN successfully disrupted the Ntn1 gene in macrophages and their precursor monocytes in vivo, ameliorating the symptoms of T2D. However, the issue of cytotoxicity still needs further optimization.

Cell penetrating peptides (CPPs) are short peptides with the ability to across cell membranes. CPPs are suitable for preclinical and clinical studies due to their low cytotoxicity compared to other vectors and their eventual degradation to amino acids.^185^ They are currently used as tools to achieve efficient Cas9 protein and sgRNA delivery.^195^, ^196^ Ramakrishna et al.^197^ conjugated Cas9 protein to CPP via a thioether bond, whereas gRNA bound with CPP to form positively charged NPs. The NPs resulted in efficient gene disruption and low off‐target mutation rates compared to plasmid transfections in embryonic stem cells, dermal fibroblasts, HEK293T cells, HeLa cells, and embryonic carcinoma cells. Moreover, Suresh et al.^196^ delivered the CRISPR–Cas9 system using CPP‐conjugated recombinant Cas9 protein and CPP‐complexed gRNAs to disrupt gene expression in human cell lines. Meanwhile, an amphiphilic penetrating peptide formed via a hydrazone bond formation between a cationic peptide scaffold and a hydrophobic aldehyde tail was designed for direct delivery of Cas9, with results showing that 30%–40% gene editing efficiency was obtained.^198^ These results have collectively indicated that CPPs have great potential for applications in CRISPR–Cas9 system. However, there are some limitations for CPPs, including a short half‐life in the blood and nonspecific delivery.^199^ Therefore, improving the specificity and efficiency of CPPs is the direction for future breakthroughs, especially in tumor cells.

Inorganic nanomaterials can also be used for vector delivery. Gold nanoparticles (AuNPs) are considered as good vectors for gene delivery as they are easy to control in terms of size and distribution, have good chemical stability and biosafety, and can be chemically modified.^200^, ^201^ Mout et al.^202^ designed cationic arginine gold nanoparticles (ArgNPs) that successfully delivered engineered Cas9 protein and sgRNA to HEK‐293T and Raw 264.7 cell lines, and achieved a 90% delivery efficiency and 30% gene editing efficiency. Furthermore, a light‐controlled release nano‐delivery system (LACP) based on AuNPs‐plasmids was developed for the delivery of Cas9‐sgPLK‐1 plasmids (CP). The results showed effective knockdown of the PLK‐1 gene in melanoma and inhibition of tumor growth in vitro and in vivo.^203^ Tao et al.^204^ were the first to report the application of gold nanoclusters (AuNCs) encapsulated with protamine as Cas9‐sgRNA plasmid vectors, and found that AuNCs were able to rapidly assemble with Cas9‐sgRNA plasmids, while the cationic protamine facilitated the efficient release of Cas9‐sgRNA plasmids in the nucleus. Therefore, AuNPs provide a safe delivery method for CRISPR–Cas components and are expected to play a greater role in the clinical treatment of tumors.

### 5.3 Other emerging nonviral delivery

Graphene oxide (GO) and black phosphorus (BP) nanosheets can also be used for CRISPR–Cas9 delivery. GO has good bio‐compatibility and safety, and is capable of loading Cas9/sgRNA RNP after modification with PEG and PEI. GO‐PEG‐PEI can rapidly transfer RNP into human cells while retaining Cas9 activity, with a gene editing efficiency of 39%.^205^ Besides, BPs, also known as isotopes of elemental phosphorus, have good biocompatibility. Zhou et al.^206^ loaded Cas9 RNP onto BP nanosheets by electrostatic adsorption. Under acidic environment, BP can be rapidly degraded to biocompatible inorganic phosphate and RNP is released into the nucleus, ultimately achieving efficient gene editing and gene silencing.

In recent years, the intrinsic properties between DNAs allow DNA nanostructures to self‐assemble in precisely controlled sizes and shapes. Currently, DNA nanostructures are considered as an emerging delivery system with the advantages of high payload capacity, good biocompatibility and biodegradability, and high stability under physiological conditions.^207^, ^208^, ^209^ Sun et al.^210^ synthesized yarn‐like DNA nanoparticles that can efficiently load Cas9/sgRNA complexes, and successfully delivered to the nucleus of human cells to achieve EGFP gene knockdown. DNA nanoparticles have not been widely used for delivery of CRISPR–Cas components, probably due to the complexity of the operation.

Apart from the abovementioned novel nonviral delivery systems, extracellular vesicles (EVs) have been shown to carry a variety of biomolecules (plasmids, siRNAs, and miRNAs) due to their high biocompatibility and low immunogenicity.^211^ In contrast to lentiviral delivery systems, EVs do not contain any viral genome. Therefore, EVs do not integrate into the host genome.^212^ In addition, EVs release Cas9 transiently in cells, greatly reducing the chance of off‐target effects due to long‐term Cas9 expression,^213^ making them the ideal system for the delivery of CRISPR–Cas9 components. Indeed, two main approaches have been reported to improve the encapsulation of CRISPR–Cas9 by EVs: engineering EVs or introducing CRISPR–Cas9‐enriched components that efficiently load mRNA and RNP into EVs through molecular interactions,^214^, ^215^, ^216^ confirming the applicability of EVs for in vivo gene therapy.

## 6 CLINICAL APPLICATION AND CHALLENGES IN CANCER

Today, CRISPR technologies have been applied to a range of scientific studies such as precision genome editing and transcriptional regulation, enabling scientists to manipulate gene sequences “at will”. In addition, CRISPR‐based gene therapies are already being used in clinical trials, particularly for diseases caused by genetic mutations.^217^, ^218^ One of the most effective ways to study the function of these mutations is to create models carrying mutated genes. Importantly, with the help of CRISPR technology, a large number of cancer models can be generated by modifying key cancer‐related genes. By developing cell‐specific viral and nonviral vectors, specific cell can be precisely manipulated, and thus cellular and animal models can be constructed.^219^ For example, several genes could be successfully modified in single mouse hematopoietic stem cells to recapitulate genetic complexity in human malignancies by delivering sgRNAs and Cas9 with a lentiviral vector, as exemplified by the creation of acute myeloid leukemia murine model through simultaneously mutating five genes in a single mouse hematopoietic stem cell.^220^ Similarly, PTEN and p53 are known as two tumor suppressor genes.^221^, ^222^ Accordingly, a murine model of liver tumors was successfully constructed by Xue et al.^223^ through delivering a CRISPR plasmid DNA expressing Cas9 and sgRNAs to liver by hydrodynamic injection to directly target and inactivate PTEN and p53 gene expressions. Moreover, Maresch et al.^224^ successfully induced pancreatic cancer models by delivering CRISPR–Cas9 vectors directly into the pancreas of mice. These studies have collectively demonstrated that CRISPR systems mediated gene editing can be efficiently used for establishing a variety of cancer models, which greatly facilitates study of molecular mechanisms of cancer and therapeutics for cancer therapy.

CRISPR systems have shown great potential not only for the construction of cancer models but also for the treatment of cancer, especially in cancer immunotherapy. When cancer cells invade, immune cells can remove them through “immune surveillance”. However, cancer cells have evolved an “immune escape” mechanism to survive. Cancer immunotherapy (also called “immuno‐oncology”) is used to enhance the anti‐cancer ability of immune cells so that cancer cells cannot complete immune escape.^225^ Anti‐programmed death 1 (PD‐1) antibodies are one of the most studied and fastest growing immunotherapies in clinical practice. In 2016, Chinese scientists conducted the world's first human CRISPR clinical trial (NCT02793856) to confirm the safety and feasibility of clinical application of CRISPR–Cas9. Specifically, researchers extracted T cells from nonsmall cell lung cancer patients and co‐transfected Cas9 and sgRNA plasmids into isolated T cells by electroporation, thereby editing the PD‐1 gene of the T cells. The amplified engineered cells were then reinfused into the patients. The results showed a median progression‐free survival of 7.7 weeks (95% confidence interval, 6.9–8.5 weeks) and a median overall survival was 42.6 weeks (95% confidence interval, 10.3–74.9 weeks) in 12 patients. In addition, the median mutation frequency of off‐target events was 0.05% (range 0%–0.25%).^218^

However, immunotherapies that block PD‐1 checkpoints have sometimes shown nonresponsiveness. Therefore, CRISPR is often used to implement cancer immunotherapy by combining synthetic biology to screen the genome. Manguso et al.^226^ screened 2368 genes in melanoma using CRISPR and identified PTPN2 as a new immunotherapy target. Upon PTPN2 knockdown, IFN‐γ‐mediated antigen presentation and growth inhibition were enhanced, further improving the efficacy of immunotherapy. Similarly, in animal models of triple‐negative breast cancer (TNBC), the immune efficacy of CD8 T cells against TNBC was enhanced following the identification of Dhx37 knockdown by CRISPR screening of CD8 T cells.^227^ Using the same approach, Wang et al.^228^ identified in TNBC mouse models that deletion of the E3 ubiquitin ligase Cop1 resulted in reduced tumor macrophage infiltration and enhanced antitumor immunity.

As the application of immune checkpoint inhibitors gradually expands, new advances in CAR‐T therapy research continue to emerge. A similar clinical trial using CRISPR gene editing T‐cell was also conducted in the U.S (NCT03399448). The researchers used CRISPR–Cas9 to knock out three genes (TRAC, TRBC, and PDCD1) from the patients' T cells, and such modified T cells (CAR‐T cells) were reinfused into three patients with refractory cancer. The results showed that the edited T cells were survived in vivo for up to 9 months without any serious adverse events, demonstrating the feasibility of CRISPR–Cas9 for cancer immunotherapy.^229^

Although autologous CAR‐T cells have shown promising results in cancer treatment, there are still some barriers to overcome, particularly the extreme high cost for personalized VAR‐T cell production. Excitingly, universal CAR‐T cells from healthy donors offer a strategy for large‐scale clinical application. In an open‐label dose‐escalation phase I study, Hu et al.^230^ developed universal CD19/CD22‐targeting CAR‐T cells (CTA101) using CRISPR/Cas9 to disrupt TRAC region and CD52 gene. After 28 days, the complete remission (CR) rate in six patients who received CTA101 infusion was 83.3%. Importantly, PD‐1 blockade could potentially enhance the immunotherapeutic efficacy of CAR T cells. It was verified that CAR T cells were able to recognize mesothelin overexpressed in human TNBC cells. Meanwhile, after knocking down the PD‐1 motif in human primary T cells using CRISPR/Cas9, CAR T cell cytokine production and cytotoxicity against PD‐L1‐expressing cancer cells were strongly enhanced. In vivo experimental results further revealed that tumor growth was significantly inhibited.^231^ Thus, combined immune checkpoint blockade with CAR T cells may enhance cancer immunotherapy and provide a strategy for controlling solid tumor progression.

Currently, three CRISPR–Cas gene editing based allogeneic CAR‐T cell therapies are in cancer clinical trials (NCT04035434, NCT03398967, and NCT03166878). In addition, enabling in vivo editing can further expand the applicability of cancer treatment. The team from the First Affiliated Hospital of Sun Yat‐Sen University piggybacked the CRIPR‐Cas9 plasmid into a gel containing C32‐447 and Poloxamer 407, which in turn disrupted the E6/E7 DNA of HPV16 and HPV18 (NCT03057912). The efficacy of in vivo editing is influenced by the precision of delivery. Fortunately, delivery strategies for CRISPR–Cas systems are being refined at an accelerated pace. Currently, CRISPR–Cas systems are already available for clinical trials of various types of cancer, and the outcome will be emerging shortly (Table 4). Collectively, the CRISPR–Cas system represents a powerful and promising tool in cancer modeling, diagnosis and treatment.

**TABLE 4:** The CRISPR‐based clinical trials for cancer treatment

Target gene	Condition or disease	Phase	Status	First Posted	ClinicalTrials.gov Identifier	Sponsor
PD‐1 and TCR	Solid tumor, adult	Phase I	Recruiting	June 4, 2018	NCT03545815	Chinese PLA General Hospital
CISH	Gastrointestinal cancers	Phase I/II	Recruiting	June 11, 2020	NCT04426669	Intima Bioscience, Inc.
E6 and E7	HPV‐related malignant neoplasm	Phase I	Unknown	February 20, 2017	NCT03057912	First Affiliated Hospital, Sun Yat‐Sen University
PD‐1	Solid tumor, adult	Phase I	Unknown	November 20, 2018	NCT03747965	Chinese PLA General Hospital
CD19	B‐cell malignancy; Non‐Hodgkin lymphoma; B‐cell lymphoma	Phase I	Recruiting	July 29, 2019	NCT04035434	CRISPR Therapeutics AG
PD‐1	Esophageal cancer	Not Applicable	Completed	March 16, 2017	NCT03081715	Hangzhou Cancer Hospital
PD‐1	Metastatic nonsmall cell lung cancer	Phase I	Completed	June 8, 2016	NCT02793856	Sichuan University
NF1	Neurofibromatosis type 1; Tumors of the central nervous system	Not Applicable	Suspended	November 6, 2017	NCT03332030	Roger Packer
CD70	T‐cell lymphoma	Phase I	Recruiting	August 6, 2020	NCT04502446	CRISPR Therapeutics AG
TP53	High grade ovarian serous adenocarcinoma	Not Applicable	Recruiting	July 30, 2018	NCT03606486	University of Washington
TGF‐β receptor II	Solid tumor, adult	Phase I	Not yet recruiting	July 26, 2021	NCT04976218	Chinese PLA General Hospital
CD19	Leukemia lymphocytic acute (ALL) in relapse; Leukemia lymphocytic acute (All) Refractory; Lymphoma, B‐cell	Phase I	Recruiting	July 30, 2019	NCT04037566	Xijing Hospital
CD5	CD5+ relapsed/refractory hematopoietic malignancies; Chronic lymphocytic leukemia (CLL); Mantle cell lymphoma (MCL); Diffuse large B‐cell lymphoma (DLBCL); Follicular lymphoma (FL); Peripheral T‐cell lymphomas (PTCL)	Early Phase I	Not yet recruiting	February 23, 2021	NCT04767308	Huazhong University of Science and Technology
BCMA	Multiple myeloma	Phase I	Recruiting	January 28, 2020	NCT04244656	CRISPR Therapeutics AG
PD‐1	Stage IV gastric carcinoma; Stage IV nasopharyngeal carcinoma; T‐cell lymphoma stage IV; Stage IV adult Hodgkin lymphoma; Stage IV diffuse large B‐cell lymphoma	Phase I/II	Recruiting	February 7, 2017	NCT03044743	Yang Yang
NTLA‐5001	Acute myeloid leukemia	Phase I/II	Recruiting	October 4, 2021	NCT05066165	Intellia Therapeutics
TCR and B2M	B‐cell leukemia; B‐cell lymphoma	Phase I/II	Recruiting	May 25, 2017	NCT03166878	Chinese PLA General Hospital
CD70	Renal cell carcinoma	Phase I	Recruiting	June 18, 2020	NCT04438083	CRISPR Therapeutics AG
CD19	Non‐Hodgkin lymphoma; Lymphoma; B‐cell lymphoma; B‐cell non‐Hodgkin's lymphoma	Phase I	Recruiting	November 20, 2020	NCT04637763	Caribou Biosciences, Inc.
CD19	Acute lymphoblastic leukemia; Chronic lymphocytic leukemia; Non‐Hodgkin lymphoma	Phase I	Not yet recruiting	September 8, 2021	NCT05037669	University of Pennsylvania
CD52 and TRAC	B acute lymphoblastic leukemia	Phase I	Recruiting	September 21, 2020	NCT04557436	Great Ormond Street Hospital for Children NHS Foundation Trust
CD19, CD20 and CD22	B‐cell leukemia; B‐cell lymphoma	Phase I/II	Recruiting	January 16, 2018	NCT03398967	Chinese PLA General Hospital
PD‐1	Advanced hepatocellular carcinoma	Phase I	Recruiting	June 5, 2020	NCT04417764	Central South University

*Note*: The information is from ClinicalTrials.gov, accessed in February 2022.

However, there are still some challenges associated with CRISPR technologies remain to be circumvented before moving to clinical applications, including adaptability, editing efficiency, delivery methods, off‐target effects, and potential on‐target mutagenesis.^232^, ^233^ For example, the p53 gene is known as the “guardian of the genome”. DNA double‐strand breaks could be recognized by the p53 gene, which in turn prevents cell division and corrects the error, thus affecting editing efficiency of CRISPR systems. It has been shown that CRISPR–Cas9 gene editing can activate the p53‐mediated DNA damage response, which increases potential risks.^234^ These issues must therefore be addressed if CRISPR systems are to be used to precisely target cancer‐related genes in human patients. Excitingly, the advent of extended toolkits and synthetic biology offers a solution to these issues. By artificially controlling various components, the behavior of molecules can be precisely regulated, allowing us to efficiently circumvent the shortcomings of CRISPR systems during cancer diagnosis and treatment. For instance, Huang et al.^235^ applied logic circuits and optogenetic devices to the split‐dCas9 system in an attempt to inhibit bladder cancer progression. The system used AND logic gates with the hTERT and hUP II promoters as activation keys. Using a similar strategy, the expression of p53 or E‐cadherin protein could be activated by induction of blue light, thereby inhibiting tumor cell proliferation. Altogether, the combined use of one or more synthetic biology strategies offers a potential therapeutic intervention for cancer treatments.

## 7 CONCLUSIONS

The CRISPR–Cas system is considered as a powerful tool for cancer treatment due to its robust gene editing capabilities. To date, various types of CRISPR–Cas systems have been developed to be suitable for treating different cancer types. Multifunctional modifications of Cas effector proteins have enhanced the targeting specificity and expanded the targeting range of CRISPR–Cas systems. Incorporating synthetic biology into the CRISPR–Cas system allows us to engineer the CRISPR–Cas systems, making them suitable for clinical application in cancer treatment. Moreover, various newly established delivery strategies made it possible to achieve specific genome editing in vivo, paving the way for successful genetic corrections in human via in vivo gene editing. A number of CRISPR‐based therapies are currently in clinical trials, and we are eagerly waiting for the outcomes to be reported shortly. It is important to note that majority of the endpoints for current ongoing clinical trials are mainly focused on safety and feasibility of CRISPR‐based therapies. More preclinical and clinical data are needed with a view to the future use of CRISPR technology for clinical treatment of a wide range of human cancers.

## AUTHOR CONTRIBUTIONS

**Xiang Meng:** Data curation (lead); investigation (lead); methodology (lead); visualization (lead); writing – original draft (lead). **Tian‐gang Wu:** Data curation (equal); investigation (equal); methodology (lead); visualization (lead); writing – original draft (equal). **Qiu‐yue Lou:** Data curation (equal); investigation (equal); methodology (equal); visualization (equal); writing – original draft (equal). **Kai‐yuan Niu:** Formal analysis (equal); investigation (equal); software (equal). **Lei Jiang:** Investigation (equal); software (equal). **Qing‐zhong Xiao:** Conceptualization (equal); project administration (equal); resources (equal); supervision (equal); writing – review and editing (equal). **Tao Xu:** Conceptualization (equal); funding acquisition (equal); project administration (equal); resources (equal); supervision (equal). **Lei Zhang:** Conceptualization (equal); funding acquisition (equal); project administration (equal); resources (equal); supervision (equal); writing – review and editing (equal).

## FUNDING INFORMATION

The present work was supported by the Natural Science Foundation of China [81700522]; the University Natural Science Research Project of Anhui Province [KJ2021A0272]; 2021 Disciplinary Construction Project in the School of Dentistry, Anhui Medical University [2021kqxkFY06]; the School Research Fund Project of Anhui Medical University [2021xkj127].

## CONFLICT OF INTEREST

The authors declare that they have no conflict of interest.

### PEER REVIEW

The peer review history for this article is available at https://publons.com/publon/10.1002/btm2.10474.

## Data Availability

The data that support the findings of this study are available from the corresponding author upon reasonable request.
